# Esophageal metastasis from hepatocellular carcinoma after orthotopic liver transplantation

**DOI:** 10.1055/a-2697-2586

**Published:** 2025-09-22

**Authors:** Songming Ding, Tian Shen, Hengkai Zhu, Yiting Hu, Shusen Zheng, Qiyong Li

**Affiliations:** 1 Division of Hepatobiliary and Pancreatic Surgery, Shulan Hangzhou Hospital Affiliated to Zhejiang Shuren University, Shulan International Medical College, Hangzhou, China

Gastrointestinal involvement in patients with hepatocellular carcinoma (HCC) is found in 0.5%–2% of cases 1 . Esophageal metastasis from HCC is seldom found, with a reported incidence of less than 0.4% 2 ; most case are determined at postmortem 3 . Here, we present an extremely rare case of premortem diagnosis of esophageal metastasis from HCC after orthotopic liver transplant (OLT).

A 60-year-old man underwent OLT for chronic liver failure associated with diffuse HCC with portal vein tumor thrombosis (PVTT). He had a history of esophageal variceal bleeding, and was treated with lauromacrogol and Histoacryl (B. Braun, Rubi, Spain). The recovery after OLT was not uneventful, with complications such as ascites, abdominal infection, pneumonia, oliguria, and anuria. The anti-rejection drug regimen was tacrolimus combined with mycophenolate mofetil and later switched to sirolimus. On postoperative day (POD) 37, a jejunal feeding tube was inserted to enhance delivery of enteral nutrition. There were no visible protrusive lesions of the esophageal mucosa ( Fig. 1 ). The alpha-fetoprotein (AFP) decreased from (>48 400 ng/mL) before OLT to 133.5 ng/mL.

**Fig. 1:**
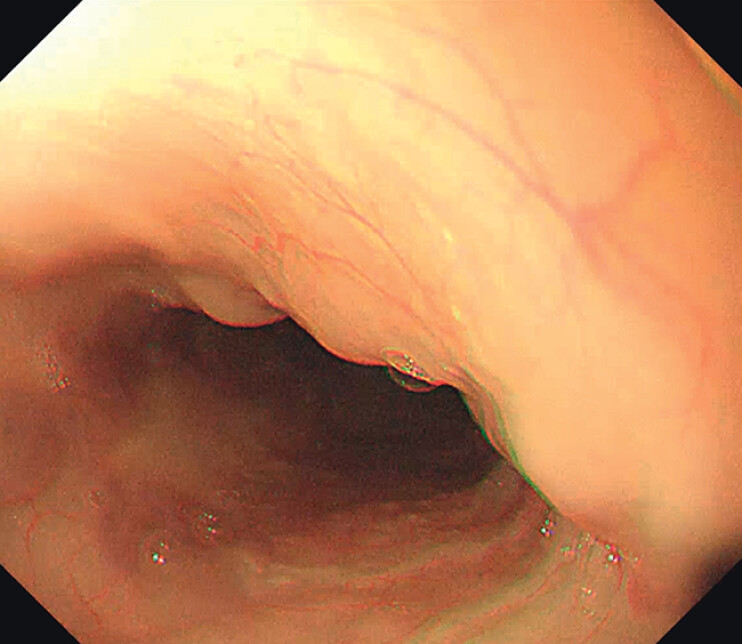
There was no visible metastatic esophageal tumor.

On POD 108, multiple polypoid masses were found in the esophagus ( Fig. 2 ). As we believed these to be benign – epithelial erosion and inflammation – no endoscopic biopsy was performed. At 5 months post-OLT, the AFP level had elevated to 2897.3 ng/mL. A whole-body positron emission tomography computed tomography (CT) scan indicated metastases of HCC to the anterior abdominal wall, 12th thoracic vertebra, and sternum ( Fig. 3 ). At 6 months post-OLT, he was readmitted because of melena and recurrent fever. Enhanced CT and magnetic resonance cholangiopancreatography showed post-transplant cholangiopathy with bile lake formation ( Fig. 4 ). Gastroscopy revealed an increase in the size and number of the protrusive lesions in the esophagus ( Video 1 ). Endoscopic biopsy pathology confirmed the multiple esophageal protrusive lesions as metastases from HCC (Glypican-3 [+], Hepatocyte [+], and AFP [+]) ( Fig. 5 ). At 7 months post-OLT, endoscopy indicated that the esophageal metastatic HCC had progressed significantly ( Video 1 ). Unfortunately, due to poor general condition, he was then unable to tolerate any antitumor treatment.

**Fig. 2:**
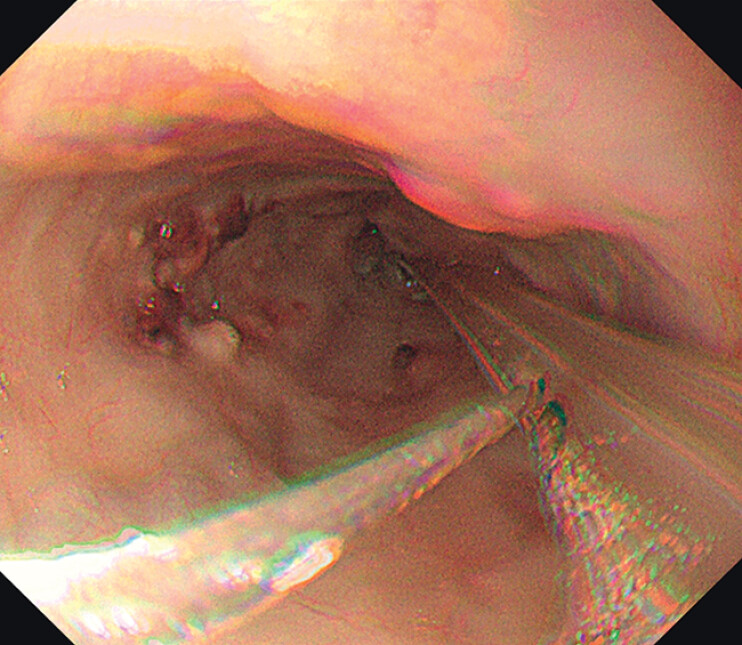
Multiple polypoid masses were observed in the esophagus.

**Fig. 3:**
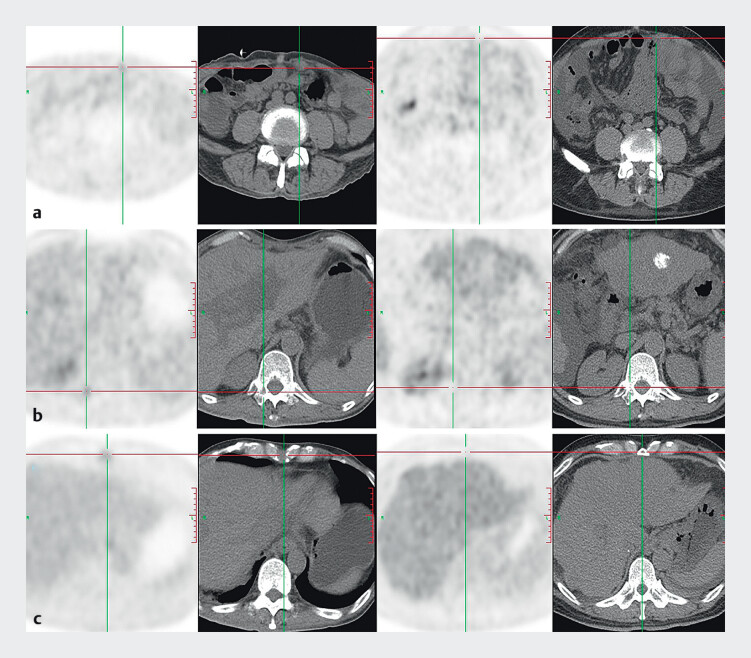
A whole-body positron emission tomography computed tomography showed metastases of **a** hepatocellular carcinoma to the anterior abdominal wall, **b** 12th thoracic vertebra, and **c** sternum.

**Fig. 4:**
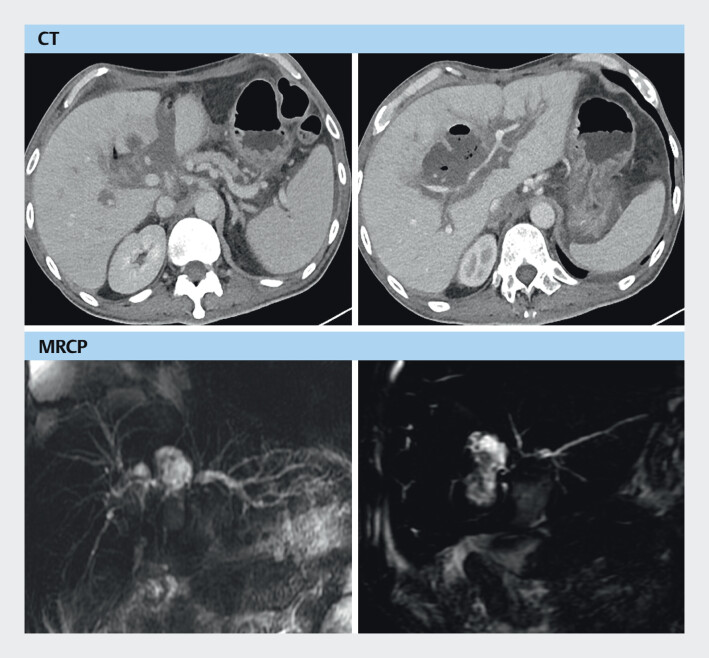
Abdominal enhanced computed tomography (CT) and magnetic resonance cholangiopancreatography (MRCP) showed post-transplant cholangiopathy with bile lake formation.

Gastroscopy examination and biopsy.Video 1

**Fig. 5:**
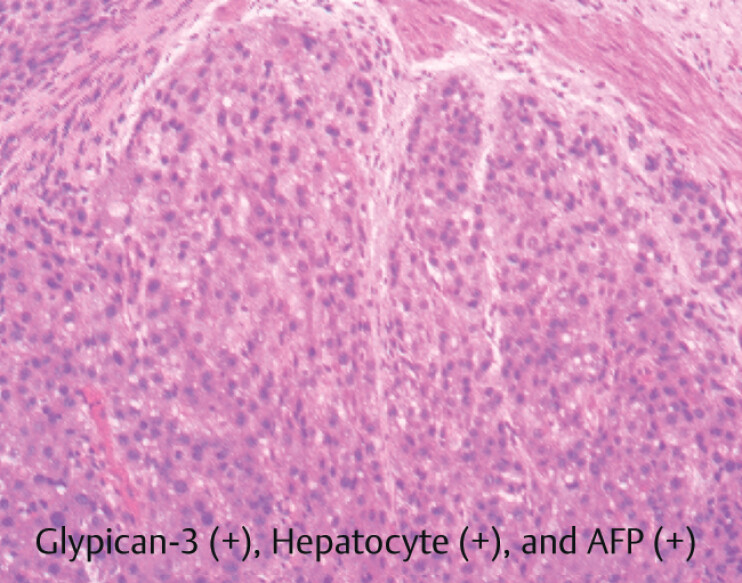
Pathological study confirmed the multiple esophageal protrusive lesions as metastatic hepatocellular carcinomas. AFP, alpha-fetoprotein.

This esophageal HCC metastasis is believed to be associated with the PVTT, via the hematogenous spreading route 4 5 . Immunosuppression may also contribute partly to the distant metastasis of HCC 3 . Although rare, esophageal HCC metastasis is possible and should be paid more attention.

Endoscopy_UCTN_Code_CCL_1AB_2AC_3AB

## Notes

Endoscopy E-Videos https://eref.thieme.de/e-videos
*E-Videos* is an open access online section of the journal *Endoscopy* , reporting on interesting cases and new techniques in gastroenterological endoscopy. All papers include a high-quality video and are published with a Creative Commons CC-BY license. Endoscopy E-Videos qualify for HINARI discounts and waivers and eligibility is automatically checked during the submission process. We grant 100% waivers to articles whose corresponding authors are based in Group A countries and 50% waivers to those who are based in Group B countries as classified by Research4Life (see: https://www.research4life.org/access/eligibility/ ). This section has its own submission website at https://mc.manuscriptcentral.com/e-videos .
